# Force-Sensor-Integrated Buckle for Wearable Robots

**DOI:** 10.3390/s26216772

**Published:** 2026-10-26

**Authors:** Heeyeon Jeong, Seran Lee, Jinhak Hwang, Seong Jun Park, Cheol Hoon Park, Hyouk Ryeol Choi, Uikyum Kim

**Affiliations:** 1 Department of Mechanical Engineering, Sungkyunkwan University, Suwon 16419, Republic of Korea; glen1493@skku.edu (H.J.); choihyoukryeol@gmail.com (H.R.C.); 2 Department of Intelligent Robotics, Sungkyunkwan University, Suwon 16419, Republic of Korea; leeseran@g.skku.edu (S.L.); jinh7736@skku.edu (J.H.); 3 Advanced Robotics Research Center, Korea Institute of Machinery and Materials (KIMM), Daejeon 34103, Republic of Korea; sjpark61@kimm.re.kr (S.J.P.); parkch@kimm.re.kr (C.H.P.)

**Keywords:** capacitance sensing, dual-axis force sensing, force sensor, sensorized buckle, wearable robots

## Abstract

Wearable robots require compact sensing interfaces to monitor the forces transmitted through attachment straps. This study aims to combine strap fastening and force measurement in a lightweight buckle, enabling the magnitude and direction of strap loading to be monitored at the attachment interface. The proposed buckle incorporates two capacitive sensing cells and a flexure structure that converts applied loads into measurable capacitance changes. Their combined responses provide dual-axis force sensing within the buckle body. The complete device measures 64×45×10mm and weighs 34g, including its sensing and signal-processing electronics. Its force and angle ranges are 0–200N and ±8°, respectively. The average capacitance sensitivity was 1.33fF/N, and the force linearity error was 6.96% of full scale of output (FSO). Force root-mean-square error (RMSE) was 7.34% of FSO without initial preload and 1.21–1.76% of FSO with approximately 18N preload in the respective tests. The validation angle RMSE was 1.67% of FSO. Force hysteresis and repeatability were 8.88% and 0.51% of FSO, respectively. The force and angle FSO values were 200N and 16°. The estimated −3dB bandwidth was 2.7Hz. The proposed design provides a compact sensing interface for strap-based wearable robots.

## 1. Introduction

Wearable robots and exoskeletons assist human movement in rehabilitation and physically demanding tasks [1]. Their assistance is transmitted through physical attachment interfaces, making the loads exchanged with the wearer an important part of human–robot interaction assessment [2]. Force sensing in these systems must be compatible with the structures that attach the robot to the body and transmit assistance. Resistive, capacitive, optical, magnetic, and pneumatic sensing approaches have been investigated for this purpose, with their installation and use depending on the load to be measured.

Wearable sensing platforms also monitor contact stimuli and body kinematics. Li et al. developed conductive starch hydrogels with state-dependent functionality: swollen hydrogels for pressure sensing and dried films for humidity sensing [3]. Zhang et al. used inductively coupled wearable loops to monitor bilateral knee flexion [4]. These studies illustrate the range of wearable measurements, while attachment-force sensing specifically characterizes the mechanical loads transmitted between a robot and its wearer.

Recent studies also show that the mechanical behavior of the attachment depends on loading direction and interface configuration. Schiebl et al. characterized nonlinear, direction-dependent stiffness at an upper-arm interface [5], while Wang et al. found different quasi-static and dynamic stiffnesses for lateral-strap and anteroposterior knee-exoskeleton attachments [6]. These findings motivate force measurements that account for the local load path and its orientation at the attachment.

Resistance-based sensing has been implemented in both contact arrays and load-bearing structures. Tamez-Duque et al. arranged force-sensitive resistors (FSRs) on the thigh and shin straps of a lower-limb exoskeleton to monitor contact-pressure distribution [7]. Ghonasgi et al. used FSR panels in an upper-limb cuff to examine distributed loads under different strap-tightness and padding conditions [8]. Choi et al. combined two FSRs with a mechanical load-transmission structure to measure hip assistance force [9]. Strain-gauge sensing has also been applied to structural deformation: Grosu et al. measured multi-axis interaction forces at a rehabilitation exoskeleton’s pelvis support [10], while Christensen et al. integrated beam-type force sensors into upper-arm and forearm cuffs [11].

Optical methods provide another way to read load-induced deformation. Optical force sensors have been incorporated into hand-exoskeleton modules for contact-force measurement [12] and developed as compact sensing units for wearable robotic systems [13]. Leal-Junior et al. embedded polymer optical fiber Bragg gratings in a flexible shank support and used their wavelength shifts to measure human–exoskeleton interaction forces during knee flexion and extension [14]. This approach places the sensing element within the support that connects the user’s limb to the robot.

Capacitive, magnetic, and pneumatic sensors have also been integrated into compliant contact interfaces. Langlois et al. used flexible capacitive pressure sensors in an upper-arm interface to estimate pressure distribution and external force during dynamic tasks [15]. Cho et al. embedded Hall-effect sensors and magnets in soft contact pads of an exosuit to measure normal and two-axis shear forces for biomechanical analysis [16]. Wang et al. measured pressure changes in soft pneumatic chambers mounted at a hip exoskeleton’s thigh interface to obtain interaction force and estimate the delivered assistive torque [17]. Hoffmann et al. embedded silicone-capsule sensor pads with piezoresistive pressure readout in an upper-body exoskeleton interface to measure normal contact loads and identify uneven loading [18]. Alagi et al. combined capacitive force sensing with shape-memory-alloy actuation in compliant interface units; their user study investigated active restoration of contact and force-signal quality after imperfect donning and movement-induced displacement [19]. These examples show how different sensing principles can be adapted to the geometry and loading conditions of a wearable interface.

Sensing can also be embedded in the elements that transmit force through a soft wearable robot. Araromi et al. developed a compliant carbon fiber–textile sensor prototype for soft exosuits, using changes in electrical contact resistance to measure tensile loads up to 300N [20]. Basla et al. integrated a stretchable silicone-based strain gauge with the passive elastic elements of an exosuit to estimate transmitted forces during walking [21]. Xie et al. developed an artificial tendon that combines tension sensing through triboelectrification and electrostatic induction with closed-loop force regulation in artificial-muscle-driven wearable robotics [22]. Slaughter et al. integrated sensing into the clutch of a quasi-passive back exosuit, estimating assistance force from encoder measurements while supporting posture monitoring and mode switching [23]. These studies illustrate how force sensing can be incorporated into the mechanical transmission path.

Related cable-driven systems also illustrate approaches to local tension measurement and model-based force control. Zhou et al. developed a miniature FSR-based tension sensor array near the distal scaffold of a cable-driven surgical robot to reduce the influence of tendon–sheath friction on tension measurements [24]. Guagliumi et al. implemented hybrid position–force control for overconstrained cable-driven parallel robots using motor torque or motor following error, together with a transmission-friction model, without force sensors in the feedback loop [25]. Although these systems serve different applications, they highlight the importance of measurement location and transmission modeling when assessing forces in a tensile load path.

The applications above use force measurements to evaluate contact loading, quantify assistance at limb supports, and monitor or regulate tension in transmission elements. Joint torque measurements likewise support muscular-torque estimation and exoskeleton control [26,27]. Gong et al. recently demonstrated a miniature piezoceramic six-axis force/torque sensor for monitoring dynamic interface-force and joint-torque variations in an exoskeleton [28]. The measurement location determines which part of the interaction is characterized: contact sensors describe loading at the body interface, whereas a sensor in an individual strap measures the force transmitted through that attachment path. Sensor integration must therefore address both the required measurement and the mechanical function of the component carrying it.

For strap-based wearable robots, the buckle is a useful integration site because it already fastens and guides the strap while carrying the transmitted load. Combining force sensing with this component can reduce the need for a separate in-line sensing unit and its mounting parts. Such a design places the measurement directly in the strap load path while retaining the fastening function needed to attach the robot to the wearer. The sensing elements and electronics must consequently fit within the buckle geometry while preserving its mechanical load path.

The fastening state is also a relevant operating variable. Lee and In evaluated an active strap for a hip orthosis that tightened according to the hip angle, examining both mechanical performance and physiological responses to periodic versus sustained tension [29]. Together with the active-interface work of Alagi et al. [19], this highlights the relevance of monitoring attachment loads as fastening conditions change. The present buckle addresses the measurement aspect; it does not actively adjust strap tension.

For such a buckle, the measurement objective includes the direction of the applied load relative to the buckle body. When strap orientation changes, the same tensile-force magnitude can produce different axial and transverse components. Measuring magnitude together with the in-plane loading angle therefore describes changes in the loading condition that a scalar tension reading alone does not resolve. A buckle that estimates these components can provide both the transmitted-force magnitude and its orientation relative to the attachment, complementing the contact-load measurements obtained elsewhere on the wearable interface.

**Figure 1:**
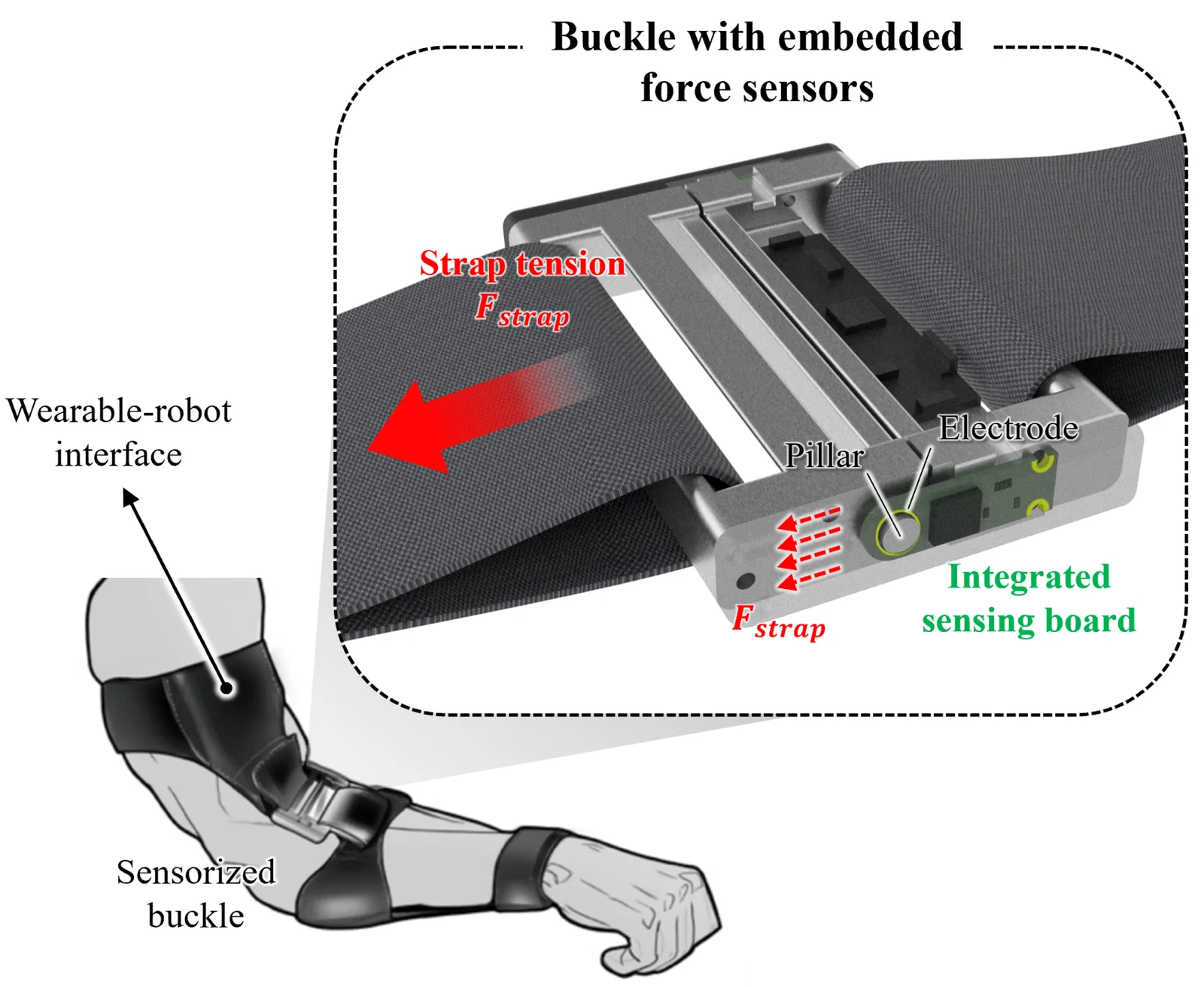
Sensorized-buckle application concept and strap-load transmission in an upper-limb wearable robot.

This paper presents a buckle-integrated capacitive force sensor, referred to as a sensorized buckle, for strap-based wearable robots. Figure 1 illustrates its proposed placement in the strap path of an upper-limb assistive system, combining mechanical fastening and force sensing. A folded aluminum flexure converts strap loading into relative motion between cylindrical pillars and annular electrodes in two sensing cells arranged bilaterally. Joint calibration of the channel outputs estimates the equivalent cell loads, from which static-equilibrium relations reconstruct the in-plane force components, total force, and loading angle.

The main contributions are (1) a compact buckle design integrating strap fastening, a folded flexure, and bilateral capacitive sensing; (2) a cell-load-based calibration and reconstruction approach for estimating strap-force magnitude and in-plane direction; and (3) experimental characterization through force and angle calibration, loading–unloading tests, and repeatability and frequency-response tests using mechanical test rigs. A quantitative comparison with existing force sensors is presented in Section 4.4 and Table 2.

## 2. Development of the Proposed Sensor

### 2.1. Sensing Principle

Capacitive force sensors convert load-induced changes in electrode geometry into capacitance changes. This principle has been used in compact wearable and multi-axis robotic force/torque sensors because the sensing electrodes can be integrated into compliant mechanical structures [30,31]. Recent miniature capacitive sensor design has also addressed multi-axis sensitivity through joint consideration of flexure geometry, electrode area, and initial electrode gap [32]. This work highlights the need to consider both the mechanical deformation and its electrical readout when integrating capacitive sensing into a small load-bearing component. Capacitive sensors detect displacement through changes in electrode overlap or separation. For parallel electrodes, normal displacement changes the separation, whereas tilting produces a spatially varying gap. Kim et al. employed eccentric cylindrical electrodes to sense shear displacement through changes in the sidewall clearance [33].

**Figure 2:**
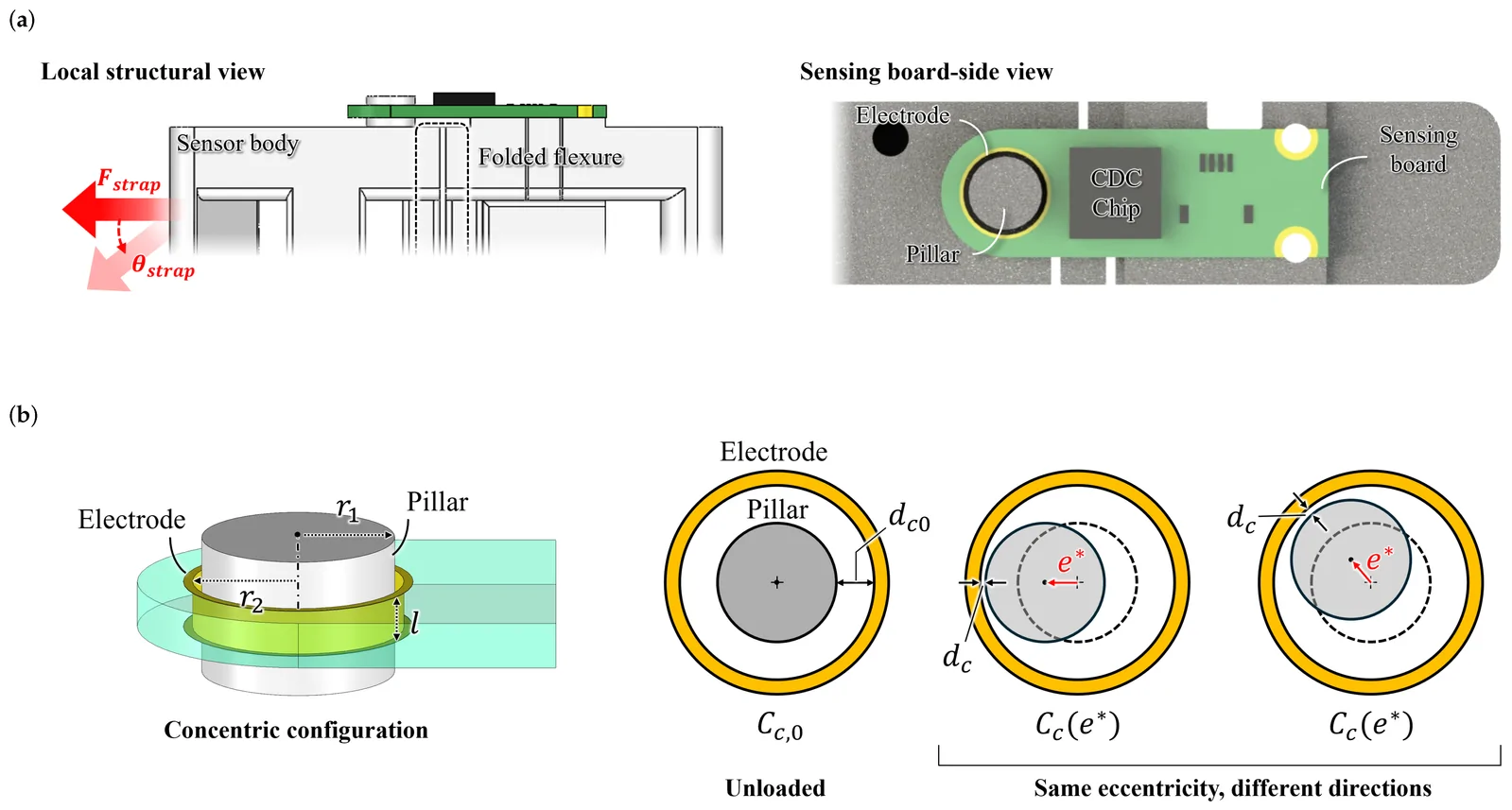
Capacitive sensing principle: (**a**) sensing structure; (**b**) concentric state and two eccentric states with equal offsets in different directions. CDC: capacitance-to-digital converter.

Building on this sensing principle, the present buckle employs a cylindrical pillar surrounded by an annular electrode. This geometry was selected to obtain a rotationally symmetric response to transverse pillar displacement. In the idealized concentric geometry, equal displacement magnitudes produce equal capacitance changes regardless of displacement direction, as illustrated in Figure 2b.

Figure 2a shows the sensing board mounted on the sensor body, with the grounded pillar positioned inside the annular electrode. The local structural view indicates the strap-loading direction and inclination, labeled $F_{\mathrm{strap}}$ and $\theta_{\mathrm{strap}}$. Strap force $F_{\mathrm{strap}}$ deforms the body’s folded flexure and displaces the pillar relative to the electrode. This relative motion changes their eccentricity and hence the capacitance measured by the capacitance-to-digital converter (CDC) on the sensing board. A sensing board is installed on each side of the body.

To describe this mechanism, Figure 2b idealizes the pillar–electrode pair as an eccentric cylindrical capacitor. Let $r_{1}$ be the pillar radius, $r_{2}$ the effective inner radius of the surrounding electrode, and *l* the effective axial overlap, with $r_{2}>r_{1}>0$. In the fabricated sensor, the gap between the pillar and annular electrode is filled with air, with no silicone or other elastomeric filler. The idealized model treats this gap as a uniform dielectric with permittivity $\varepsilon_{0}\varepsilon_{r}$, where $\varepsilon_{0}$ is the vacuum permittivity and $\varepsilon_{r}\approx 1$ is the relative permittivity of air, and neglects end effects and fringing fields. The cylindrical surfaces are assumed to have parallel axes and a constant axial overlap. This idealization explains the sensing mechanism; it does not include the full three-dimensional field around the finite printed circuit board (PCB) electrode.

In Figure 2b, the dashed circles indicate the unloaded pillar position. The unloaded pair is concentric, and loading produces a relative center-displacement vector e in the plane perpendicular to the pillar axis. Its magnitude *e* is the distance between the pillar and electrode centers, while the axial overlap *l* remains fixed. The initial radial clearance $d_{c0}$, eccentricity magnitude *e*, and minimum loaded clearance $d_{c}$ are related by

(1) $$d_{c0}=r_{2}-r_{1},\;e=∥e∥,\;d_{c}=d_{c0}-e.$$

Along the line joining the two centers, displacement reduces the minimum clearance to $d_{c0}-e$ and increases the clearance on the opposite side to $d_{c0}+e$. The noncontact model applies for $0\leq e<d_{c0}$. In the concentric state (e=0), the capacitance is

(2) $$C_{c,0}=\frac{2\pi \varepsilon_{0}\varepsilon_{r}l}{\ln\left(r_{2}/r_{1}\right)}.$$

For $0<e<d_{c0}$, we follow the bipolar-coordinate solution for eccentric cylindrical capacitors given by Morales et al. [34]. The pillar surface and the inner surface of the annular electrode correspond to constant coordinates $\tau_{1}$ and $\tau_{2}$, respectively, with $\tau_{1}>\tau_{2}>0$. Solving Laplace’s equation with constant electrode potentials and integrating the charge over the full circumference gives

$$C_{c}=\frac{Q}{V}=\frac{2\pi \varepsilon_{0}\varepsilon_{r}l}{\tau_{1}-\tau_{2}},$$

where *Q* is the charge magnitude and *V* is the magnitude of the potential difference between the electrodes. The circle geometry relates the coordinate difference to the radii and eccentricity through

$$\cosh\left(\tau_{1}-\tau_{2}\right)=\frac{r_{1}^{2}+r_{2}^{2}-e^{2}}{2r_{1}r_{2}}.$$

Combining these relations yields the idealized eccentric capacitance

(3) $$C_{c}=\frac{2\pi \varepsilon_{0}\varepsilon_{r}l}{\mathrm{arccosh}\left(\frac{r_{1}^{2}+r_{2}^{2}-e^{2}}{2r_{1}r_{2}}\right)}.$$

At e=0, the denominator becomes $\ln(r_{2}/r_{1})$, so (3) reduces to the concentric expression in (2). The model predicts increasing capacitance as the minimum clearance decreases.

The capacitance variation caused by loading is therefore

(4) $$\Delta C_{c}=C_{c}-C_{c,0}.$$

The red arrows in the two loaded states of Figure 2b represent transverse pillar displacements of the same magnitude e* but different directions. These states therefore have equal ideal capacitances $C_{c}\left(e*\right)$.

Let the transverse displacement be expressed as $e=\left(e_{x},e_{y}\right)=\left(e\cos\phi ,e\sin\phi \right)$, where ϕ is its direction in the plane perpendicular to the pillar axis. Equation (3) depends only on $e^{2}=e_{x}^{2}+e_{y}^{2}$ and contains no dependence on ϕ. Therefore, rotating the displacement direction while keeping its magnitude fixed leaves the ideal capacitance unchanged. This symmetry describes the ideal displacement-to-capacitance response of an individual cell; it does not imply an identical response to equal strap forces applied in different directions. The strap-force magnitude and direction are reconstructed from the jointly calibrated responses of the two cells and the buckle mechanics. Residual misalignment and departures from the ideal electrode geometry may introduce directional dependence in the fabricated cell.

For the fabricated buckle, $r_{1}=2.0\;\mathrm{mm}$ and $r_{2}=2.3\;\mathrm{mm}$, giving $d_{c0}=0.3\;\mathrm{mm}$. The eccentric-cylinder expression accounts for the nonuniform clearance produced by transverse displacement. The geometry allows the annular electrode to surround the pillar within the side-mounted sensing board, integrating capacitive readout into the buckle structure.

**Figure 3:**
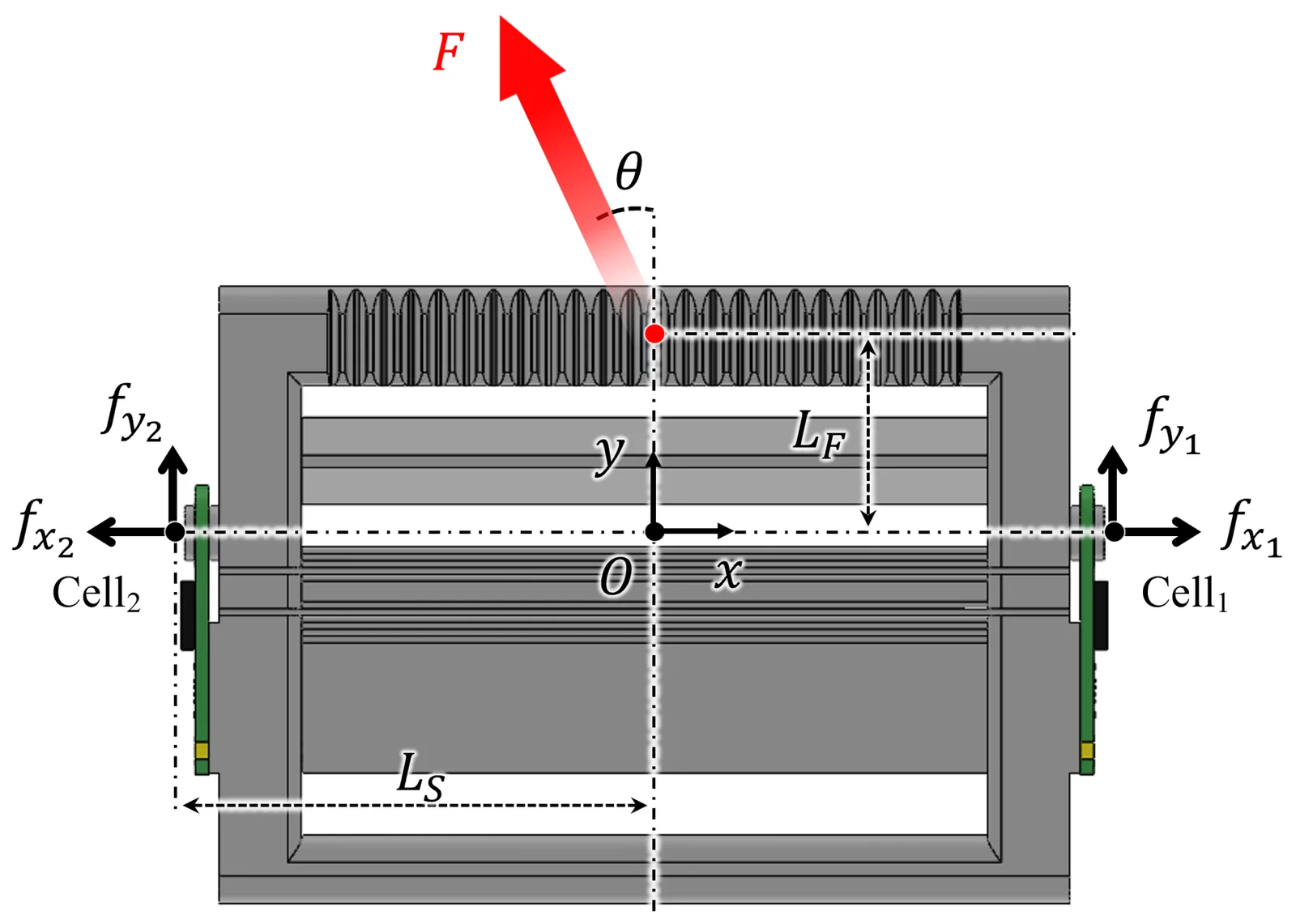
Geometric relationship between the strap force and equivalent reaction forces at the two pillar locations.

Figure 3 shows the strap force *F* and the force components $f_{xi}$ and $f_{yi}$ at each pillar (i=1,2), in the directions indicated by the arrows. The reference point *O* is the midpoint between the two pillars. Here, $L_{S}$ is the distance from *O* to either pillar, $L_{F}$ is the axial distance from *O* to the strap-force application point, and θ is the loading angle from +y toward −x. Force balance and moment balance about *O* give

(5) $$\begin{array}{cc} f_{x1}-f_{x2}+F_{x} & =0,\\ f_{y1}+f_{y2}+F_{y} & =0,\\ L_{S}\left(f_{y1}-f_{y2}\right)-L_{F}F_{x} & =0. \end{array}$$

For calibration, define tensile-positive equivalent cell loads by $q_{1}=-f_{y1}$ and $q_{2}\;=\;-f_{y2}$, and let $\gamma =L_{S}/L_{F}$. The applied force components in the coordinates of Figure 3 are defined as

(6) $$\begin{array}{cc} F_{x} & =-F\sin\theta ,\\ F_{y} & =F\cos\theta . \end{array}$$

Here, $F_{x}$ and $F_{y}$ are the transverse and axial components of the strap force, respectively. Substituting the cell-load definitions into Equation (5) yields

(7) $$\begin{array}{cc} F_{x} & =\gamma (q_{2}-q_{1}),\\ F_{y} & =q_{1}+q_{2}. \end{array}$$

An aligned load has $q_{1}=q_{2}$, while a positive loading angle produces $q_{1}>q_{2}$ in this model. The total force and loading angle follow as

(8) $$F_{\mathrm{total}}=\sqrt{{\left(q_{1}+q_{2}\right)}^{2}+{\left[\gamma \left(q_{1}-q_{2}\right)\right]}^{2}}.$$

(9) $$\theta =atan2\left[\gamma \left(q_{1}-q_{2}\right),q_{1}+q_{2}\right].$$

Here $F_{\mathrm{total}}=F$ for the planar resultant, and atan2 preserves the sign and quadrant of its direction for nonzero force. The cell-load sum determines the axial component $F_{y}$, while their difference determines the transverse component $F_{x}$. The force-balance equations are linear in the equivalent cell loads; however, a linear relationship between the CDC outputs and these loads is not assumed. The cell loads are obtained using a second-order polynomial calibration fitted to experimental data, as described in Section 4.2, and are subsequently used for force reconstruction.

### 2.2. Flexure Design

**Figure 4:**
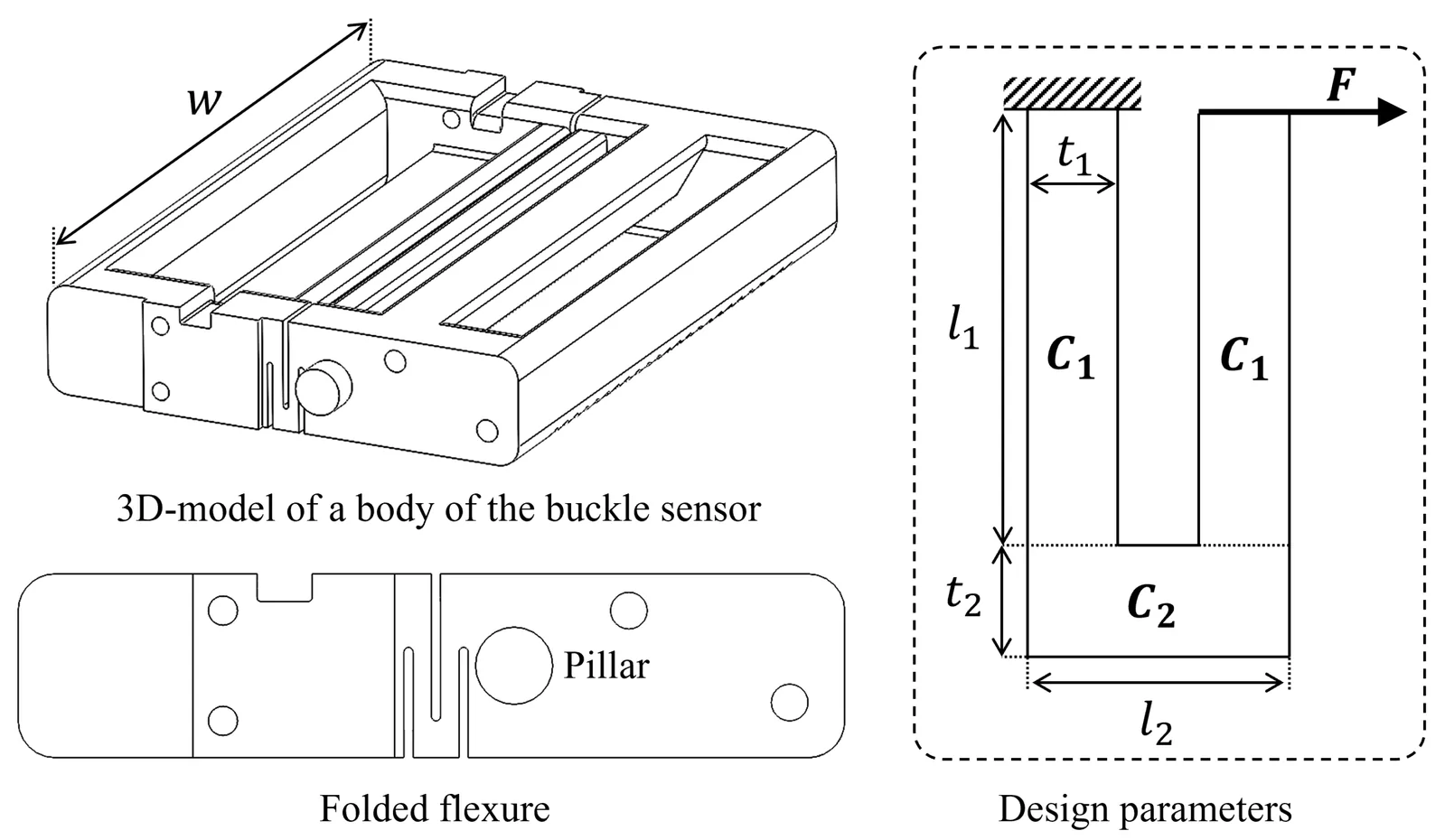
Buckle-body geometry, folded flexure and pillar arrangement, and the idealized folded-beam model with its design parameters.

A folded flexure was designed at the center of the sensor body, as shown in Figure 4, to convert strap tension into repeatable elastic displacement. The folded flexure provides compliant motion in the loading direction within the available buckle volume. Its deformation generates the relative pillar–electrode motion required for sensing while the surrounding frame carries the strap load.

For the analytical estimate, the flexure was idealized as two identical rectangular legs joined by a transverse connector, with small, linear-elastic deformation in the plane of Figure 4. The two legs are labeled $C_{1}$ and the connector is labeled $C_{2}$. Each leg has length $l_{1}$ and in-plane width $t_{1}$, while the connector has span $l_{2}$ and in-plane depth $t_{2}$. All members share the out-of-plane thickness *w*, indicated by the dimension arrow in the three-dimensional view of Figure 4. These members form a single folded load path between the fixed and loaded ends. Under the assumptions of small displacements and rotations and linear-elastic material behavior, equilibrium is evaluated using the undeformed geometry, with constant stiffness $k_{t}$ in $\delta =F/k_{t}$. Large-rotation effects and material plasticity are excluded.

The equivalent stiffness is defined as $k_{t}=F/\delta$, where δ is the displacement of the loaded end relative to the fixed end in the direction of the applied force *F*. Its reciprocal, $1/k_{t}=\delta /F$, is the compliance, or displacement per unit force. The force acts transversely to the legs, causing bending and shear deformation. The connector also deforms through axial loading and bending moments transmitted by the legs. Their contributions to the loading-direction displacement are combined in the compliance model.

The calculation uses the folded-beam compliance expression reported by Wai-Chi et al. [35]. Their reference system contains four identical branches acting in parallel, with $k_{e}=4k_{1/4}$, where $k_{1/4}$ is the stiffness of one branch. Modeling the buckle flexure as one such branch gives $k_{t}=k_{1/4}=k_{e}/4$ and hence $1/k_{t}=4/k_{e}$. This conversion accounts for the number of parallel branches. Using the dimensions defined in Figure 4, the adopted compliance estimate is

(10) $$\frac{1}{k_{t}}=\frac{4}{wE}\left(\frac{l_{1}^{3}}{2t_{1}^{3}}+\frac{6\left(1+\mu \right)l_{1}}{5t_{1}}+\frac{l_{2}}{2t_{2}}+\frac{3l_{1}^{2}l_{2}}{2t_{2}^{3}}\right),$$

where *E* is Young’s modulus and μ is Poisson’s ratio. The four terms inside the parentheses account, respectively, for leg bending, leg shear, connector axial deformation, and connector bending. The factor 1+μ in the shear term arises from the relation G=E/[2(1+μ)], where *G* is the shear modulus. The final term includes $l_{1}^{2}$ because the moment transmitted to the connector depends on the leg length. The connector geometry therefore also contributes to the predicted compliance.

Equation (10) relates the flexure dimensions to its load-to-displacement response. Increasing $l_{1}$ increases compliance, whereas increasing $t_{1}$ or $t_{2}$ reduces the corresponding bending contribution through the inverse-cubic terms. Increasing *w* or *E* increases stiffness in direct proportion. The resulting stiffness estimate was approximately $1.56\times 10^{3}\;N/\mathrm{mm}$. Using $\delta =F/k_{t}$, the predicted displacement under a 200N load was approximately 0.13mm.

**Figure 5:**
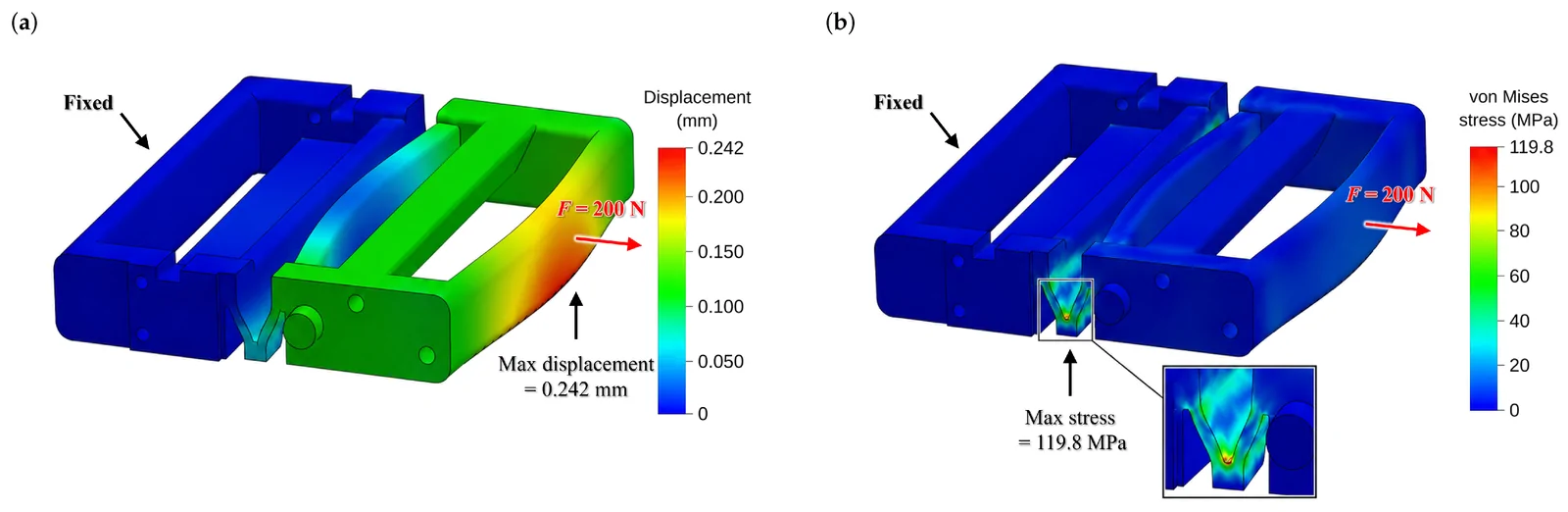
Finite element analysis under a 200N tensile load: (**a**) resultant displacement; (**b**) von Mises stress.

Finite element analysis (FEA) was performed in SolidWorks 2020 to compare the analytical stiffness estimate with the response of the complete buckle geometry. The 6061 aluminum-alloy body was fixed at the left strap-side region, and a 200N tensile load was applied to the right strap-side region. As shown in Figure 5a, displacement increased toward the loaded side, while deformation of the central folded flexure produced the relative pillar–electrode motion used for sensing.

The FEA gave a relative loading-direction displacement of approximately 0.145mm between the strap-side reference locations, corresponding to $k_{\mathrm{FEA}}≃1.38\times 10^{3}\;N/\mathrm{mm}$. The analytical model uses the simplified folded-flexure geometry shown in Figure 4, whereas the FEA model represents the more complex geometry of the complete buckle. Geometric details and local deformation represented in the FEA but omitted from the simplified analytical model may contribute to the approximately 12% difference between the two predictions. The maximum resultant displacement, approximately 0.242mm, occurred on the outer loaded frame. The stiffness comparison uses relative displacement between the strap-side reference locations, rather than the maximum resultant displacement of the frame. This comparison is between two models; experimental stiffness validation requires a force–displacement measurement at the corresponding reference locations. The current experimental setup lacks suitable instrumentation to directly measure the small relative displacement between these locations on the buckle. Therefore, experimental stiffness validation was not performed in this study and remains a task for future work.

Figure 5b shows stress concentrations near the folded-flexure connections and fixed regions, with relatively low stress across most outer-frame and strap-guiding regions. The maximum von Mises stress was approximately 119.8MPa at the highlighted inner bend/root of the folded flexure.

## 3. Structure Design and Fabrication

### 3.1. Design of the Proposed Sensor

**Figure 6:**
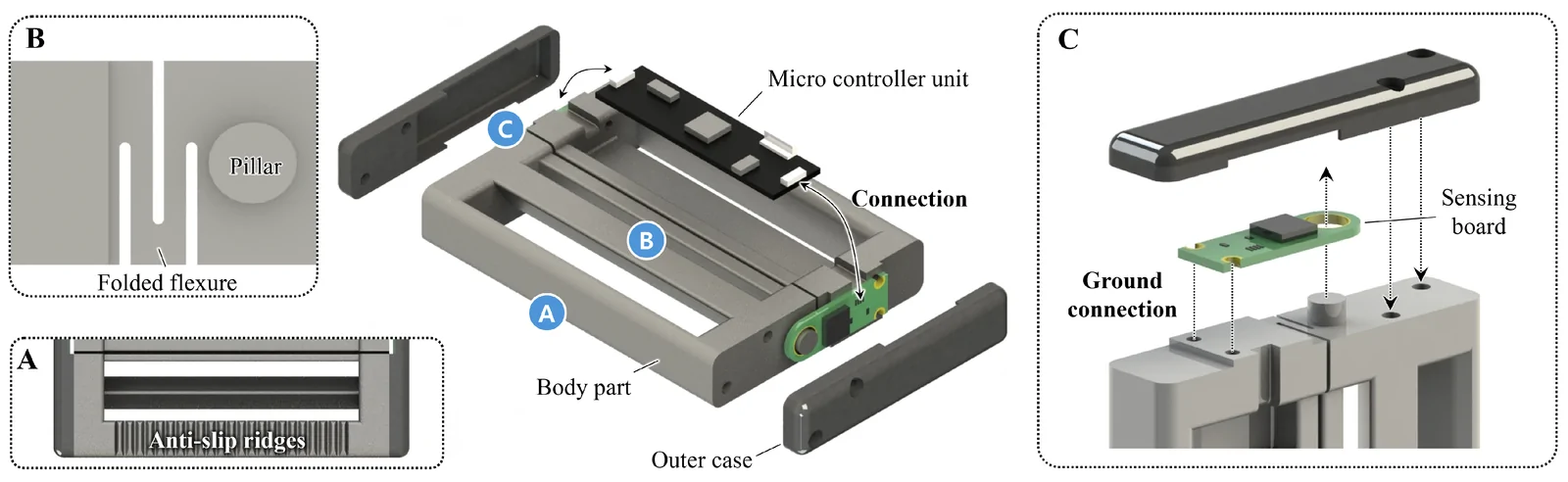
Exploded view of the sensorized buckle: (**A**) bottom view showing the anti-slip ridges; (**B**) folded flexure at the center of the sensor body; (**C**) sensing board assembly.

The sensorized buckle adopts a conventional buckle form for direct integration into a wearable-robot strap. The central exploded view in Figure 6 shows its 6061 aluminum-alloy body, microcontroller unit (MCU) board, side sensing board, outer cases, and electrical interconnections. One sensing board is installed on each side of the body, with one visible in the exploded view. The body fastens the strap and carries the applied strap load. The sensing boards detect deformation-induced capacitance changes, the MCU acquires and transmits the channel outputs, and the outer cases protect the side-mounted boards from contact and impact.

The buckle body includes strap slots and a central folded flexure. Anti-slip ridges on the lower strap-contact surface are intended to limit strap slip and the resulting changes in the loading geometry assumed by the sensing model.

The folded flexure acts as the load-to-displacement transduction structure. A cylindrical pillar is formed on each side of the body and serves as a grounded electrode facing the annular electrode on the corresponding sensing board. Flexure deformation changes the relative pillar–electrode geometry and hence the capacitance of each cell. The bilateral arrangement produces two channel responses whose asymmetry varies with loading direction; these responses are used to estimate the two equivalent cell loads.

Each sensing board contains an annular electrode, a CDC chip, conductive traces, and electrical connectors. The electrode surrounds the body pillar and is nominally concentric with it in the unloaded state. The MCU board is mounted on the upper surface of the body and connected to the sensing boards through flexible printed circuit boards (FPCBs).

The board placement shown in Figure 6C is designed to establish and maintain nominal concentric alignment between the annular electrode and the pillar in the unloaded state. The sensing board is secured at the board-to-body grounding points. The conductive body and pillar are connected to board ground, whereas the surrounding annular electrode remains electrically isolated from the pillar. This common reference is intended to reduce sensitivity to changes in external contact, nearby conductive objects, and wiring conditions. Residual manufacturing and assembly offsets can affect the unloaded capacitance and displacement sensitivity. An initial center offset $e_{0}$ makes the idealized loaded eccentricity $∥e_{0}+\Delta e∥$, where Δe is the load-induced relative displacement. Baseline subtraction removes the unloaded output offset, but does not generally remove the sensitivity change. The calibration in Section 4.2 characterizes the assembled sensor’s output–load relationship.

### 3.2. Fabrication of the Proposed Sensor

**Figure 7:**
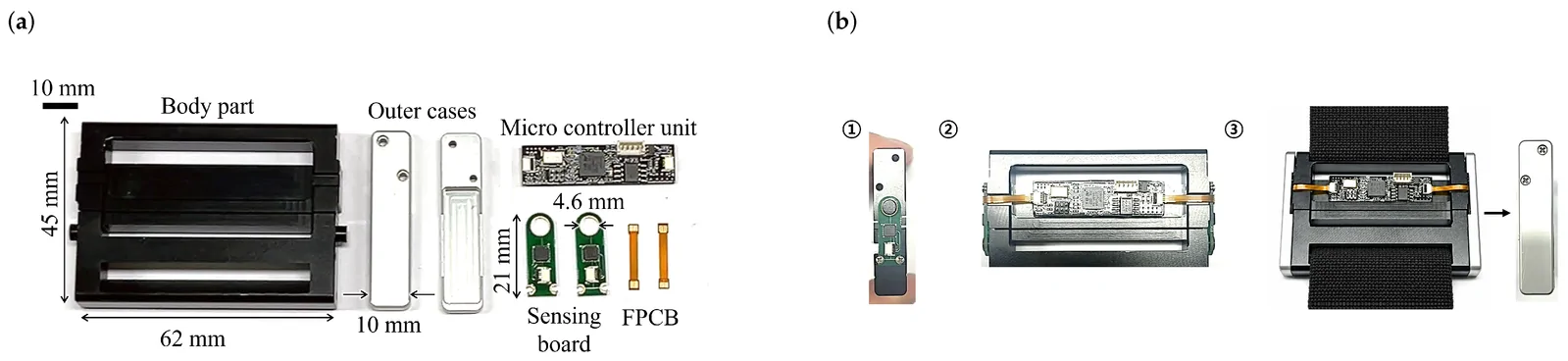
Sensorized buckle fabrication: (**a**) components and dimensions; (**b**) assembly sequence. FPCB: flexible printed circuit board.

Figure 7a presents the fabricated buckle body, two outer cases, MCU board, two sensing boards, and FPCBs. The body was fabricated from 6061 aluminum alloy and measured 62mm×45mm; each outer case was 10mm wide. Each sensing board measured approximately 21mm×7mm and incorporated an AD7147 capacitance-to-digital converter to digitize capacitance changes. Its 4.6mm-diameter opening was surrounded by an annular electrode. Together with the 4.0mm-diameter pillar, this geometry provided a nominal initial radial clearance of 0.3mm. Capacitance was measured using the AD7147, a sigma–delta capacitance-to-digital converter with an internal sampling frequency of 250kHz and a resolution of 16 bits [36]. The initial and maximum measured capacitances were approximately 0.398pF and 0.663pF, respectively. The corresponding maximum capacitance change was 0.265pF (265fF), representing an increase of approximately 66.6% relative to the initial capacitance.

Figure 7b presents three stages of assembly. The first view shows a sensing board secured to the side of the body, with its annular electrode surrounding the pillar. The second shows the MCU board mounted on the upper surface of the body and connected to both sensing boards through FPCBs. The final view shows the assembled buckle with the strap and outer cases in place. The cases were fastened to the body with M1.8 bolts to protect the side-mounted electronics.

The completed device measured approximately 64mm×45mm×10mm and weighed approximately 34g. It integrates the buckle body, capacitive sensing cells, signal-conversion circuitry, MCU, and communication interface within a buckle-shaped form factor. Its physical characteristics and experimentally evaluated performance are summarized in Table 1.

## 4. Performance Evaluation

### 4.1. Experimental Setup

**Figure 8:**
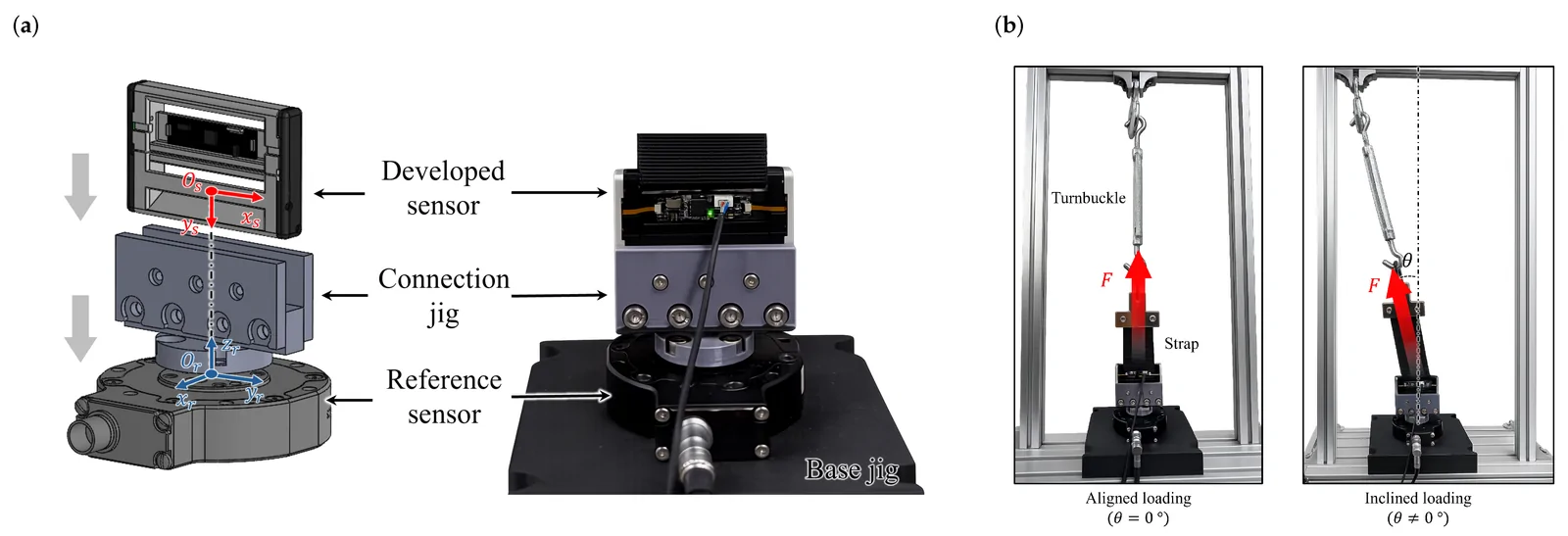
Calibration setup: (**a**) sensor mounting and coordinate frames; (**b**) aligned and inclined strap loading.

Calibration experiments were conducted to relate the two CDC outputs to the applied strap load. As shown in Figure 8a, the developed sensor was mounted above the six-axis force/torque reference sensor (AFT200, AIDIN Robotics) through a connection jig, and the reference sensor was fixed to a base jig. The sensor frame $\left(O_{s},x_{s},y_{s}\right)$ and reference frame $\left(O_{r},x_{r},y_{r},z_{r}\right)$ were registered by the fixed jig geometry.

Tensile load was applied through a strap connected to the frame by a turnbuckle, as shown in Figure 8b. In the aligned configuration, the load was parallel to the sensor’s longitudinal axis; inclined loading introduced a transverse component. The developed-sensor and reference-sensor signals were recorded simultaneously. The reference-sensor outputs were expressed in the sensor coordinate frame used in Figure 3. The +y axis in Figure 3 and the $+y_{s}$ axis in Figure 8a are physically aligned. The calibration force components follow the axes and sign conventions in Figure 3. Unloaded offsets were removed before calibration.

### 4.2. Calibration Model and Estimation Performance

**Figure 9:**
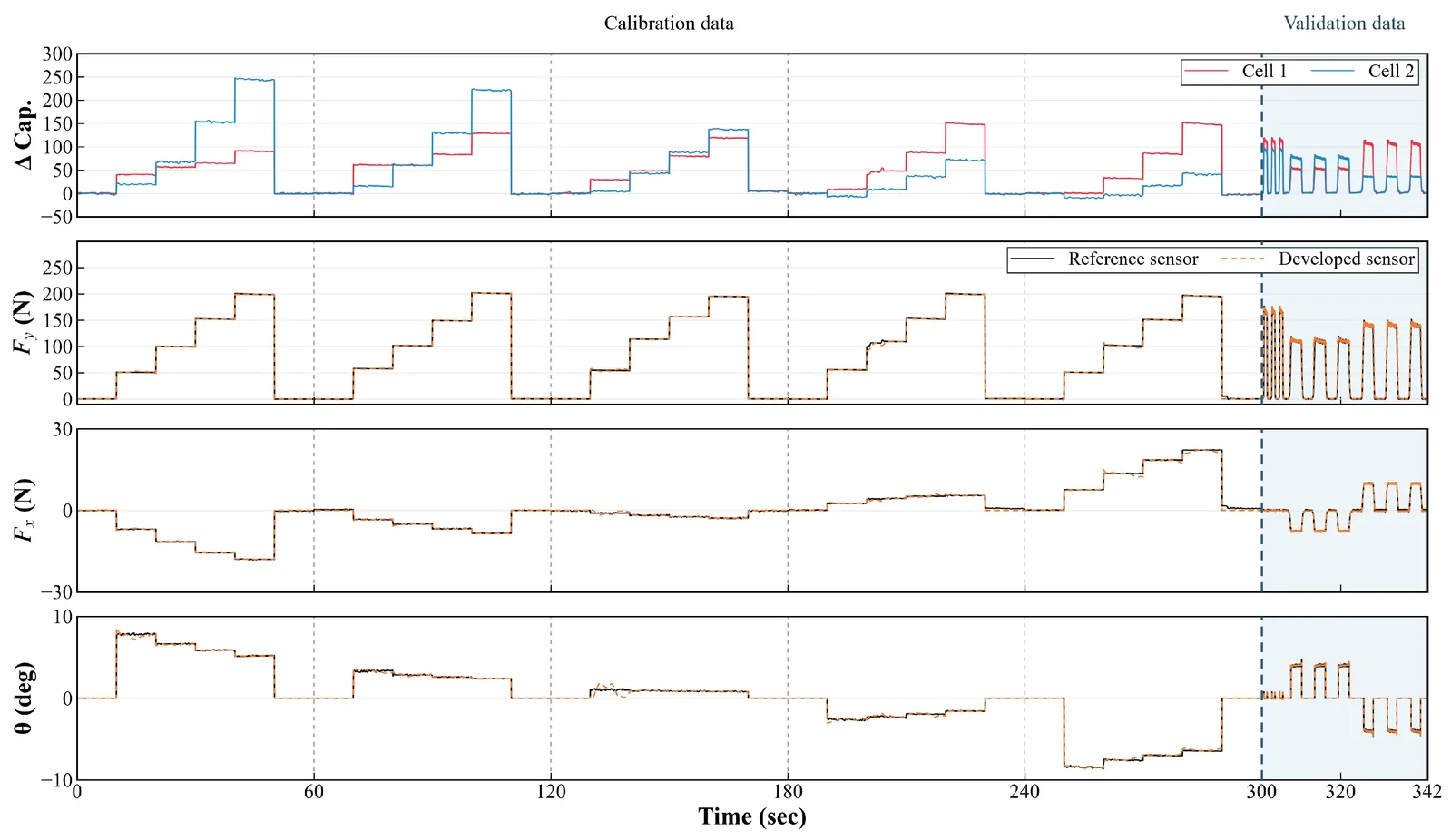
Sensor calibration and validation results. Δ Cap.: change in capacitance digital counts.

For calibration, the digitized CDC output of each sensing cell was referenced to its unloaded baseline. The output variations of Cell 1 and Cell 2 were defined as

(11) $$\Delta C_{i}=C_{i}-C_{i,0},\;i=1,2,$$

where $C_{i}$ denotes the digitized CDC output, expressed in counts, and $C_{i,0}$ is the corresponding unloaded baseline for each experimental trial. The baseline-referenced outputs form the calibration inputs, and the calibration portion of Figure 9 shows the channel responses in counts. Here, $\Delta C_{i}$ denotes a digital output change, whereas $\Delta C_{c}$ in Section 2.1 denotes a physical capacitance change. The count-valued responses should not be interpreted as capacitance values in farads without a verified conversion. The tensile-positive equivalent cell loads are defined as $q_{1}=-f_{y1}$ and $q_{2}=-f_{y2}$, as introduced in Section 2.1. Using the force components measured by the reference sensor and Equation (7), the reference equivalent loads of Cell 1 and Cell 2 were calculated as $q_{1,\mathrm{ref}}=\frac{1}{2}\left(F_{y,\mathrm{ref}}-F_{x,\mathrm{ref}}/\gamma \right)$ and $q_{2,\mathrm{ref}}=\frac{1}{2}\left(F_{y,\mathrm{ref}}+F_{x,\mathrm{ref}}/\gamma \right)$, where $\gamma =L_{S}/L_{F}=1.2335$ was determined from the computer-aided design (CAD) model. The equivalent load of each cell is estimated using a second-order polynomial calibration that includes both baseline-referenced CDC outputs, their squared terms, and their cross term:

(12) $$\begin{array}{cc} {\hat{q}}_{i}= & \beta_{i,0}+\beta_{i,1}\Delta C_{1}+\beta_{i,2}\Delta C_{2}\\ \; & +\beta_{i,3}\Delta C_{1}^{2}+\beta_{i,4}\Delta C_{1}\Delta C_{2}\\ \; & +\beta_{i,5}\Delta C_{2}^{2},\;i=1,2. \end{array}$$

The calibration coefficients $\beta_{i,k}$ were determined for each cell by least-squares fitting to the reference equivalent cell loads calculated from the reference force measurements, using data from all five loading sequences in the 0–300s calibration portion of Figure 9. Additional cyclic-loading responses are displayed at 300–342s. The additional record used for quantitative force validation below was excluded from coefficient fitting and evaluated using the unchanged coefficients. Both channels and their cross term account for channel sensitivity and interaction. The force components, total force, and loading angle follow from the estimated cell loads using Equations (7)–(9).

Figure 9 presents the cell-output changes, force components, and loading angle for the calibration and validation records. In the calibration portion, the loading orientation progresses from positive inclination through near alignment to negative inclination. Each sequence, separated by a vertical dashed line, contains four increasing axial-force plateaus of approximately 50, 100, 150, and 200N, followed by unloading. This arrangement shows how the channel responses and transverse force vary with strap orientation over similar axial-force levels.

Under positive inclination, $F_{x}$ was negative and θ was positive. Cell 1 initially showed the larger output change, while Cell 2 increased more strongly with axial load and became dominant at the higher plateaus. This difference was most pronounced at the larger positive inclination. As the strap approached alignment, the magnitude of the transverse force decreased and the channel responses became closer, with Cell 1 remaining larger at lower loads and Cell 2 becoming slightly larger at the upper load levels.

With negative inclination, $F_{x}$ became positive while $F_{y}$ remained positive. Cell 1 showed the larger output throughout the loaded plateaus, and its separation from Cell 2 became more pronounced at the larger negative inclination. The magnitude of the transverse force also increased with axial load. These responses depend on loading direction, load magnitude, and channel sensitivity. The joint calibration maps the two channel responses to the equivalent cell loads.

The estimated force components in the calibration portion of Figure 9 follow the stepped reference responses during loading and unloading. The estimated angle likewise tracks the transition from positive to negative inclination through near alignment.

The shaded 300–342s interval in Figure 9 shows a separate cyclic-loading validation record, acquired with the buckle remounted before each loading cycle. The calibration and validation records are concatenated for display.

The maximum measured capacitance change was 0.265pF over the force measurement range of 0–200N. Dividing this change by the force span yielded an average capacitance sensitivity of approximately 1.33fF/N, as summarized in Table 1.

**Figure 10:**
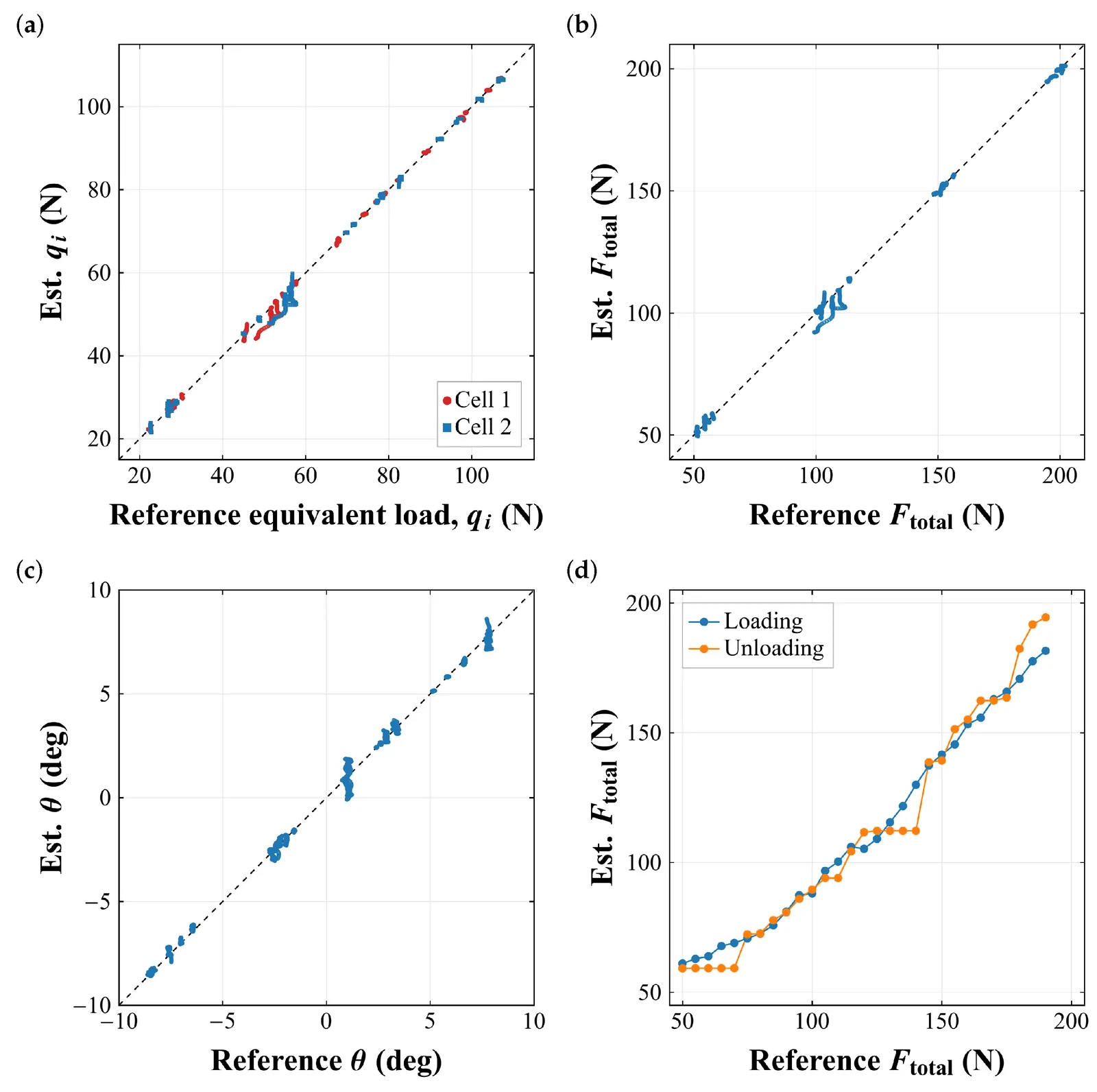
Calibration and hysteresis results: (**a**) equivalent cell loads; (**b**) total force; (**c**) loading angle; (**d**) loading–unloading response.

Figure 10a–c shows the relationship between the estimated and reference quantities within the five calibration sequences in the 0–300s portion of Figure 9. The comparison included the four loaded intervals in each sequence, including the initial response at each load step. Total force and loading angle were calculated for each sample pair. The equivalent cell loads were obtained by applying the inverse of Equation (7) to the reference and estimated force components, respectively. These plots describe the calibration data; quantitative force-estimation error is reported for the separate validation record below.

The total-force root-mean-square error (RMSE) was evaluated by comparing the validation data obtained using the existing calibration coefficients with the reference sensor measurements. The evaluation covered 32 loading cycles, including transitions, load-holding intervals, and unloaded intervals. Reference and estimated total force were recorded simultaneously at 1000Hz. This validation dataset was excluded from coefficient fitting and is distinct from the cyclic-loading trace in Figure 9. The total-force RMSE measured on the validation data was 3.54N, corresponding to a normalized RMSE of 1.77% of the 200N full scale. Angle error was evaluated separately by comparing the reference and developed-sensor angles recorded in the validation portion of Figure 9, using all intervals with a reference resultant force of at least 50N. The angle RMSE was 0.268°, corresponding to 1.67% of the 16° span from −8° to +8°. Table 1 reports this angle RMSE and the force RMSEs for the initial-tension comparison in Section 4.3, normalized by their respective spans.

Figure 10a compares equivalent cell loads over approximately 20–110N, Figure 10b compares total force, and Figure 10c compares loading angle over approximately ±8°. The dashed lines in these panels indicate identity.

The approximately ±8° range describes the local strap-force direction relative to the buckle. Exoskeleton joints and passive self-alignment mechanisms can accommodate limb motion [37]; an upper-arm cuff study also selected elbow flexion–extension to minimize upper-arm motion [8]. When the buckle and local strap load path rotate together, their relative angle is unchanged, so large joint excursions do not necessarily require an equally large loading-angle range. The evaluated range has potential for monitoring small transverse deviations in aligned fastening straps, with its adequacy depending on initial alignment and relative changes in the local load path. The cited studies do not establish universal sufficiency of ±8°. Larger relative deviations would require assessment of electrode clearance, flexure motion, and the associated change in capacitance sensitivity, followed by calibration and validation over the wider force–angle domain.

Figure 10d compares the estimated total force during loading and unloading at matched reference forces. For this cycle, the maximum loading–unloading difference in estimated force was 17.76N. Dividing this difference by the 200N full scale and multiplying by 100% gives a force hysteresis of 8.88%.

The linearity error was evaluated from the mean of the loading and unloading curves in Figure 10d. At each of the 29 matched reference forces $F_{j}=50,55,\ldots ,190\;N$, the mean output was calculated as ${\bar{\hat{F}}}_{j}=\left({\hat{F}}_{\mathrm{load},j}+{\hat{F}}_{\mathrm{unload},j}\right)/2$. A straight line $aF_{j}+b$ was fitted to these 29 mean outputs by ordinary least squares with equal weights. The resulting slope and intercept were a=0.9522 and b=−1.379N. The linearity error was defined as

(13) $$L_{F,\mathrm{mean}}=\frac{\max_{j=1,\ldots ,29}\left|{\bar{\hat{F}}}_{j}-\left(aF_{j}+b\right)\right|}{200\;N}\times 100\%.$$

The maximum deviation was 13.92N, giving $L_{F,\mathrm{mean}}=6.96\%$. This result characterizes the mean force curve for the cycle in Figure 10d over 50–190N. It does not include the separation between the two branches, which is reported separately as the 8.88% force hysteresis, and is not an angular linearity metric. The fitted line was used only to calculate the deviation; the force calibration was not refitted. This cycle-specific result does not establish linearity over all tested angles or the full 0–200N design range.

The measured hysteresis may arise from strap–buckle contact friction, time-dependent strap deformation and recovery, and small relative movements at fastened joints or sensing-board mounts. Cyclic hysteresis and time-dependent deformation have been reported for woven polyamide straps [38]. Contact friction and possible viscoelastic recovery could alter the contact state and load-transmission geometry, while movement at joints or board mounts could change the pillar–electrode position. These mechanisms could produce different capacitance outputs at the same reference force during loading and unloading. Their individual contributions were not isolated in this study.

### 4.3. Repeatability and Frequency-Response Evaluation

**Figure 11:**
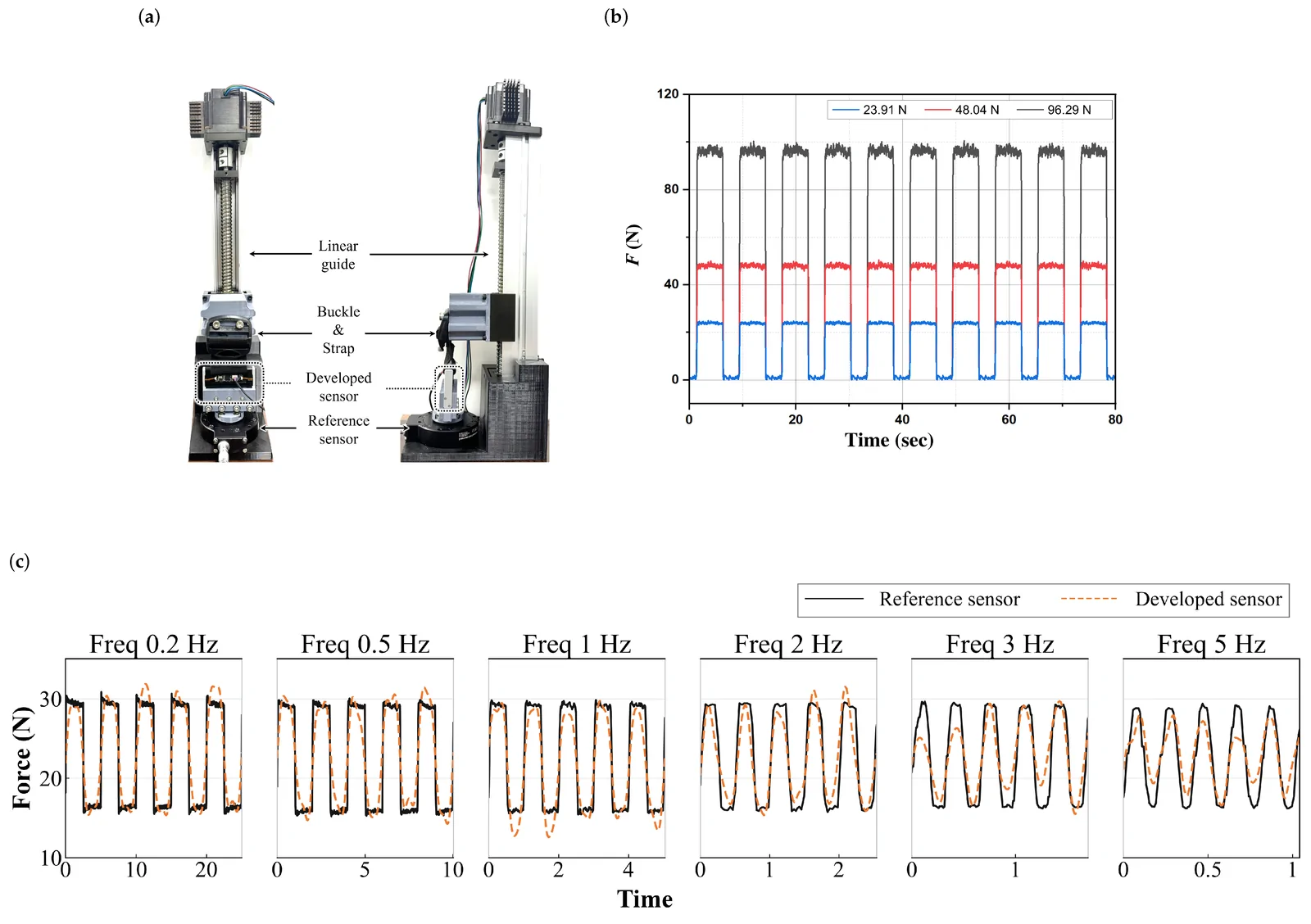
Repeatability and frequency-response tests: (**a**) linear-guide setup; (**b**) ten loading–unloading cycles at each of three force levels. (**c**) Frequency response.

The sensor was mounted in the motorized linear-guide test rig shown in Figure 11a to evaluate repeatability. The developed sensor was mounted above the reference sensor and connected by a strap to the moving carriage. Repeated motion of the guide applied and released strap tension, with ten loading–unloading cycles at each of three loading levels. Figure 11b shows stable loading plateaus and return toward baseline after unloading. The mean plateau values were 23.91, 48.04, and 96.29N. The cycle-to-cycle coefficients of variation of the plateau means were 1.14%, 1.08%, and 1.06%, respectively, remaining close to 1% at all three loading levels.

For each force level, the coefficient of variation is the standard deviation of the ten cycle-mean plateau values divided by their mean, multiplied by 100%. Normalizing the same standard deviations by the 200N full scale of output (FSO) gives approximately 0.14%, 0.26%, and 0.51% of FSO, respectively. Table 1 reports the maximum, 0.51% of FSO. These metrics characterize short-term consistency of cycle means, not within-cycle noise or long-term stability. The ten-cycle tests do not establish long-term drift, temperature sensitivity, durability, or fatigue life.

Additional cyclic-loading tests evaluated the force response at commanded frequencies of 0.2, 0.5, 1, 2, 3, and 5Hz while maintaining the same reciprocating displacement excursion. The same calibration coefficients were used for all conditions. The developed-sensor and reference force signals were recorded simultaneously. The median repetition frequencies calculated from the recorded timestamps were 0.200, 0.500, 1.001, 2.004, 3.003, and 4.955Hz, respectively.

Figure 11c illustrates force tracking using the five consecutive cycles with the lowest force RMSE in each record. Time is shown in seconds and reset for each window. The quantitative analysis used all 19 complete cycles in each record and the original force samples at their recorded timestamps.

The fundamental amplitude of each force signal was obtained by fitting a constant, sine, and cosine at the measured cycle frequency, with sample weights proportional to the represented time intervals to account for unequal timestamp spacing. The median ratio of developed-sensor to reference amplitude was normalized to its value at 0.2Hz. The resulting relative gains were 0.00, −1.02, −1.13, −1.61, −3.53, and −3.92dB across the six conditions. The −3dB crossing occurred between the 2 and 3Hz conditions, with an estimated bandwidth of approximately 2.7Hz. The sample-based force RMSE ranged from 2.42 to 3.52N. These results characterize the tested cyclic-loading conditions.

Although the estimated bandwidth of approximately 2.7Hz is relatively low, the sensor is intended to monitor slowly varying wearer forces transmitted through the attachment strap. The low-frequency tracking results support its suitability for this monitoring purpose. Future work will investigate ways to increase the bandwidth through improvements in the mechanical sensing structure and signal acquisition, followed by performance evaluation under more dynamic wearable conditions.

Measurement performance following repeated attachment and release was assessed using the nine validation cycles in Figure 9. Each cycle involved attaching the buckle, applying the load, and releasing the buckle. Force and angle RMSEs were calculated for each cycle. Force RMSE includes the complete cycle, including transitions, whereas angle RMSE uses all intervals with a reference resultant force of at least 50N. Cycles with similar loading patterns were compared in groups of three. The force RMSE ranges for cycles 1–3, 4–6, and 7–9 were 26.47–26.64N, 11.63–11.73N, and 11.29–11.49N, respectively. The corresponding within-group differences between the maximum and minimum RMSEs were 0.17, 0.10, and 0.20N. The angle RMSE ranges were 0.044–0.060°, 0.159–0.402°, and 0.123–0.259°, with within-group spreads of 0.016°, 0.243°, and 0.136°, respectively. These values describe the variation in measurement error following repeated attachment under the tested loading conditions.

To examine the relationship between initial strap tension and measurement error, the validation record in Figure 9 was compared with the preloaded frequency-response tests in Figure 11c. The initial reference force was defined as the median over the first 0.2s of each validation cycle or frequency-test record. It ranged from 0.12 to 0.27N in the validation cycles, whose pooled total-force RMSE was 14.67N (7.34% of FSO) over all nine complete cycles. In the frequency-response tests, the nominal initial tension was approximately 18N, and the recorded initial reference force ranged from 16.47 to 16.86N. The force RMSEs at 0.2, 0.5, 1, 2, 3, and 5Hz were 2.79, 2.56, 3.52, 2.42, 2.95, and 2.93N, respectively, evaluated over all 19 complete cycles per condition using the original recorded force values. Normalized by the 200N force FSO, these RMSEs ranged from 1.21% to 1.76% of FSO, as summarized in Table 1. The lower RMSEs in the preloaded tests suggest that the strap tension state may influence measurement error, highlighting the importance of controlling initial tension and mounting conditions. Loading amplitudes, rates, and mounting protocols also differed between the tests, so this comparison does not isolate the effect of initial tension. The 14.67N result characterizes the repeated-mounting record in Figure 9 and is distinct from the 3.54N force-validation result reported in Section 4.2.

The relevance of mounting conditions is consistent with recent interface studies. Active compliant units have been used to restore contact and improve force-signal quality following donning errors and interface displacement [19], and knee-exoskeleton attachment geometry has been shown to influence quasi-static and dynamic stiffness [6]. These studies concern body-contact interfaces, whereas the present measurements concern a buckle in the strap load path. They motivate future wearable validation with controlled strap tension and documented attachment geometry, but do not establish the cause of the RMSE differences observed in the present bench tests.

### 4.4. Comparison with Existing Sensors

Table 2 compares the proposed buckle with three force sensors for wearable and rehabilitation robots in terms of sensing principle, size/mass, reported performance, and features. Error definitions and test conditions differ among the studies. Dimensions and masses follow each source’s reported device scope; Cho et al.’s mass is per sensing unit, whereas the buckle mass includes its sensing and signal-processing electronics.

The principal advantage of the proposed design is the integration of strap fastening, force measurement, and loading-direction estimation in one component. Using the buckle itself as the sensing structure can reduce the need for a separate in-line force sensor and dedicated mounting parts in the strap load path. Reconstruction of two in-plane force components provides both the transmitted-force magnitude and its direction relative to the buckle, allowing changes in loading direction to be distinguished even at the same force magnitude. The sensing cells, signal-processing circuitry, and communication interface are integrated into a 64×45×10mm device weighing 34g. This combination provides a compact sensing interface at the point of strap attachment while retaining the buckle’s mechanical fastening function.

**Table 2:** Comparison with existing force sensors.

Study	Principle	Size/Mass	Reported Performance	Features
Grosu et al. [10]	Strain gauge	80×96mm/340g	Linearity: 0.2/0.28/6N (X/Y/Z)Hysteresis: 0.4% (Z)	3-axis force; pelvis interface
Wang et al. [17]	Pneumatic	NR/15g	Calibrated dynamic error: 10.3±6.58% Hysteresis: NR	Thigh force; estimated assistive torque
Cho et al. [16]	Magnetic (Hall)	15×20×5mm/4g per unit	Error/hysteresis: NR	Normal/shear force; soft contact pad
**This work**	Capacitive	64×45×10mm/34g	Force RMSE (no preload): 7.34% of FSOForce RMSE (preloaded): 1.21–1.76% of FSOHysteresis: 8.88% of FSO	Strap fastening; in-plane force/direction; integrated electronics

Not reported (NR) indicates information not reported in the main article; root-mean-square error (RMSE); full scale of output (FSO), 200N for this work.

Recent integrated sensing approaches provide complementary functions. The instrumented clutch of Slaughter et al. estimates exosuit assistance while enabling engagement control [23]; the piezoceramic sensor of Gong et al. measures dynamic multi-axis load variations at exoskeleton interfaces and joints [28]. The latter’s alternating-current-coupled measurement chain reports dynamic changes rather than absolute quasi-static loads. In comparison, the present buckle estimates strap-force magnitude and in-plane direction over the evaluated loading conditions while providing mechanical fastening. These different measurement objectives and sensing locations should be considered when assessing integration and performance.

## 5. Conclusions

This study developed a sensorized buckle that combines strap fastening with dual-axis capacitive force sensing for wearable robots. A folded aluminum flexure and two sensing cells arranged bilaterally convert strap loading into changes in the channel outputs. Joint calibration and static-equilibrium relations reconstruct the axial and transverse force components, enabling estimation of the transmitted-force magnitude and in-plane loading direction. The design integrates these measurements with mechanical attachment in a 64×45×10mm, 34g device, including its sensing and signal-processing electronics, with a 0–200N design load range.

Independent validation using unchanged calibration coefficients yielded a total-force RMSE of 3.54N, or 1.77% of the 200N full scale, over 32 loading cycles, including transitions and unloaded intervals. The force hysteresis was 8.88% of the 200N full scale in the evaluated loading–unloading cycle. Repeatability tests at three loading levels yielded a maximum cycle-mean standard deviation of approximately 0.51% of the 200N FSO, indicating consistent mean force responses across repeated cycles under the tested conditions. Cyclic-loading tests at 0.2–5Hz yielded an estimated −3dB bandwidth of approximately 2.7Hz, relative to the response at 0.2Hz. These results support the integrated sensing concept under the evaluated bench conditions.

The reconstruction assumes a defined strap-force application geometry. Strap slip, material, initial tension, and mounting changes can alter this geometry or load transmission. The repeated-mounting evaluation quantified cycle-wise force and angle errors, and the preloaded tests showed lower force RMSEs (1.21–1.76% of FSO) than the near-zero-initial-force validation record (7.34% of FSO). This comparison supports attention to initial tension and mounting conditions, although their individual effects were not isolated. The anti-slip ridges are intended to limit slip; quantitative slip measurements and comparisons among strap materials remain future work. Further assessment should compare a fixed calibration with recalibration over a wider range of loading and mounting conditions.

Further evaluation should quantify output drift under sustained loading, changes in zero offset and sensitivity with temperature, and changes in sensing performance and structural integrity under extended cyclic loading. Future work will investigate increasing the bandwidth through improvements in the mechanical sensing structure and signal acquisition, followed by validation under more dynamic wearable conditions. Further measurements should characterize the reference sensor’s dynamic response over a wider frequency range. Experimental force–displacement measurements are also needed to validate the stiffness models. These assessments will define the operating conditions for prolonged and dynamic use in wearable robots.

## Figures and Tables

**Table 1:** Sensor specifications.

Item	Specification
Size	64×45×10mm
Mass	34g
Force range	0–200N
Angle range	±8°
Sensitivity	1.33fF/N
Linearity	6.96% of FSO
Force RMSE (no preload)	7.34% of FSO
Force RMSE (∼18N preload)	1.21–1.76% of FSO
Angle RMSE	1.67% of FSO
Hysteresis	8.88% of FSO
Repeatability	0.51% of FSO
Bandwidth	2.7Hz

Root-mean-square error (RMSE); full scale of output (FSO): force, 200N (0–200N); angle, 16° (−8° to +8°).

## Data Availability

The data supporting the findings of this study are available from the corresponding author upon reasonable request.
